# PROLONGED GRIEF DISORDER IN ADULTS OVER 65: A REVIEW IN LIGHT OF POST-COVID-19 LOSSES

**DOI:** 10.1093/geroni/igae098.3062

**Published:** 2024-12-31

**Authors:** Hannah Friedland, Ashley Stripling, Jillian Crocker

**Affiliations:** Nova Southeastern University, Fort Lauderdale, Florida, United States; Nova Southeastern University, Fort Lauderdale, Florida, United States; Nova Southeastern University, Fort Lauderdale, Florida, United States

## Abstract

Prolonged Grief Disorder (PGD) entered the DSM-5-TR in 2022, standardizing criteria and research. As COVID-19 continues to disproportionately affect adults over 65, both directly and by disrupting traditional grieving rituals, understanding PGD in this group is essential. The review seeks to synthesize existing research on PGD in adults over 65 to guide research and practice. A PRISMA review utilizing “older adults” and “prolonged grief disorder” was conducted within APA PsychInfo and Google Scholar from 2013 to 2023. All English-language journal articles relating to the area of study were read, and relevant exclusion criteria were applied (i.e., empirical, mean age >65, prolonged grief symptoms for >1 year). Among the 15 articles meeting inclusion, PGD prevalence rates ranged between 0.5% and 21%. Participants were predominantly White women recruited from community and hospice samples. PGD risk factors included preexisting depression and type of relationship with the deceased, with the highest incidence rates following partner or child deaths. Co-occurring symptoms included sleep disturbances, lower quality of life, loneliness, and dysregulated cortisol. Given PGD’s addition to the DSM and inconsistent prevalence rates, accurate diagnosis and effective treatment are imperative, particularly for more impacted groups. Notably, death rates from COVID-19 are disproportionate among age, race, and ethnicity. As a true understanding of how PGD manifests in adults over 65, future research is warranted following the COVID-19 pandemic. Implications of the current presentation include translating findings into recommendations focused on the provision of adapted PGD interventions and assessments for adults over 65.

